# A guide for optimal iodine staining and high‐throughput diceCT scanning in snakes

**DOI:** 10.1002/ece3.7467

**Published:** 2021-06-26

**Authors:** Sean Callahan, Jenna M. Crowe‐Riddell, Ramon S. Nagesan, Jaimi A. Gray, Alison R. Davis Rabosky

**Affiliations:** ^1^ Museum of Zoology University of Michigan Ann Arbor MI USA; ^2^ Department of Biology Eastern Michigan University Ypsilanti MI USA; ^3^ Ecology and Evolutionary Biology University of Michigan Ann Arbor MI USA; ^4^ Florida Museum of Natural History University of Florida Gainesville FL USA

**Keywords:** anatomy, computed‐tomography, education, herpetology, imaging, morphology, museum collections, Peruvian Amazon

## Abstract

Diffusible iodine‐based contrast‐enhanced computed tomography (diceCT) visualizes soft tissue from micro‐CT (µCT) scans of specimens to uncover internal features and natural history information without incurring physical damage via dissection. Unlike hard‐tissue imaging, taxonomic sampling within diceCT datasets is currently limited. To initiate best practices for diceCT in a nonmodel group, we outline a guide for staining and high‐throughput µCT scanning in snakes. We scanned the entire body and one region of interest (i.e., head) for 23 specimens representing 23 species from the clades Aniliidae, Dipsadinae, Colubrinae, Elapidae, Lamprophiidae, and Viperidae. We generated 82 scans that include 1.25% Lugol's iodine stained (soft tissue) and unstained (skeletal) data for each specimen. We found that duration of optimal staining time increased linearly with body size; head radius was the best indicator. Postreconstruction of scans, optimal staining was evident by evenly distributed grayscale values and clear differentiation among soft‐tissue anatomy. Under and over stained specimens produced poor contrast among soft tissues, which was often exacerbated by user bias during “digital dissections” (i.e., segmentation). Regardless, all scans produced usable data from which we assessed a range of downstream analytical applications within ecology and evolution (e.g., predator‐prey interactions, life history, and morphological evolution). Ethanol destaining reversed the known effects of iodine on the exterior appearance of physical specimens, but required substantially more time than reported for other destaining methods. We discuss the feasibility of implementing diceCT techniques for a new user, including approximate financial and temporal commitments, required facilities, and potential effects of staining on specimens. We present the first high‐throughput workflow for full‐body skeletal and diceCT scanning in snakes, which can be generalized to any elongate vertebrates, and increases publicly available diceCT scans for reptiles by an order of magnitude.

## Graphical abstract

We present the first high‐throughput workflow for full‐body skeletal and soft‐tissue (dice) CT scans in preserved snakes. These methods increase publicly available diceCT scans for reptiles by an order of magnitude and can be generalized to any elongate vertebrates.

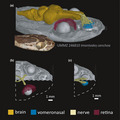

## 1 INTRODUCTION

Museum collections are foundational to studies in ecology and evolutionary biology because they create a permanent record of how organisms respond to changing environmental, climatic, and ecological forces (Lister et al., 2011). Access to collections was historically limited to those with the means to visit a museum in person. The recent revolution to digitize museum data has begun “unlocking” these collections and democratizing data on a global scale (Hedrick et al., 2020). These digitization initiatives produce great innovation in both education and research (Bakker et al., 2020), with new applications across biology, especially morphology through nondestructive specimen imaging (Gray et al., 2019; Paluh et al., 2020). However, the most commonly used imaging technology (microcomputed tomography or μCT) only detect mineralized features (e.g., bones and teeth) with limited capacity for visualizing soft‐tissue anatomy, which are vital data for understanding integrated organismal systems.

Diffusible iodine‐based contrast‐enhanced μCT (diceCT) enhances contrast of soft tissues by submerging or injecting preserved specimens with an iodine solution prior to scanning (Gignac & Kley, 2014; Gignac et al., 2016; Metscher, 2009). Postscanning, the iodine solution can be removed via leaching or chemical destaining, which has led to diceCT gaining popularity as a non‐ to minimally destructive technique (see Early et al., 2020; Hedrick et al., 2018). In addition to digital imaging of soft tissues in three dimensions (3D), diceCT can also provide access to ecological data or “natural history bycatch” that includes diet records of both hard‐ and soft‐bodied prey, parasite loads, and clutch sizes or stages of reproductive development. The combination of traditional μCT and emerging diceCT techniques can create integrative datasets for museum specimens (e.g., Clement et al., 2015; Fabbri et al., 2017), which can be shared widely and used to address questions of both form and function in biology.

DiceCT has great potential to propel comparative morphological studies forward (Gignac & Kley, 2018), but the systematic collection of diceCT data is currently limited in this field. Taxonomic representation among vertebrates is lacking; data are biased toward mammals with a narrow representation of nonmodel organisms within reptiles, amphibians, and birds (Gignac et al., 2016, references therein). A lack of taxon‐specific protocols, as well as an underreporting of diceCT successes/failures, is likely hindering progress in diceCT techniques (Gignac et al., 2016). To increase available diceCT datasets, we need a guide to initiate best practices for streamlined data generation and curation that is tailored to specific taxonomic groups as has been done for traditional μCT methods (e.g., see “scan all fishes,” Buser et al., 2020).

Snakes are an ecologically diverse clade of limbless squamate reptiles with ~3,879 species currently recognized from 20 families (Uetz, 2020). Snakes have the largest range of body sizes in any tetrapod clade besides mammals, with adults ranging from 10 cm to 9 m in length depending on the species. Snakes have been foundational to research on extreme phenotypes, especially their morphological and ecological adaptations for prey capture, physiology, locomotion, and sensory specializations (Lillywhite, 2014). Recent nondestructive imaging in snakes includes studies in locomotion (Capano, 2020), skull and fang morphology (Da Silva et al., 2018; du Plessis et al., 2018), neural and sensory systems (Gignac & Kley, 2018; Macrì et al., 2019), and previously unknown cephalic vasculature (Palci et al., 2019). DiceCT datasets (head only) have been published for just three snakes: an annulated sea snake (*Hydrophis cyanocinctus*), a western diamondback rattlesnake (*Crotalus atrox*), and a European viper (*Vipera berus*) (Gignac et al., 2016; Palci et al., 2019). Together, these studies can enhance our understanding of the ecology and evolution of transitions to elongate forms, as well as the broad diversification processes that follow these transitions.

In this study, we diceCT scanned 23 species of snakes with the following goals: (a) Determine the optimal packing and iodine staining procedure to visualize soft tissues in a taxonomically diverse set of snakes encompassing a range of body and head sizes, (b) devise an efficient workflow for high‐volume scanning of specimens that is optimized for longevity of digital specimens with minimal damage to physical specimens, and (c) assess the range of downstream applications made possible by making these data available to the scientific community. We contextualize this workflow in relation to project timelines, data sharing, and future high‐throughput diceCT studies in snakes and other underrepresented taxa, especially their potential use across diverse research and educational initiatives.

## 2 MATERIALS AND METHODS

### 2.1 Specimen selection and preservation

We stained and scanned a single specimen each from 23 species (*n* = 23 individuals) in the snake clades Aniliidae, Dipsadinae, Colubrinae, Elapidae, Lamprophiidae, and Viperidae (following nomenclature in Pyron et al., 2013; Table 1). Specimens encompassed a range of body sizes: snout to vent length (SVL) between 104 mm and 1,840 mm, and body mass between 8.4 g and 1,250 g. Specimens were sourced from the University of Michigan Museum of Zoology (UMMZ) and Museo de Historia Natural de la Universidad Nacional Mayor de San Marcos (MUSM). They had been previously fixed in 10% formalin, preserved in 75% ethanol (EtOH), and stored at UMMZ, Ann Arbor, Michigan, USA. The majority of specimens were collected during trips to Peru and Nicaragua from 2016 to 2019, and euthanized and fixed 24 hr after capture. All field collection protocols were approved by the University of Michigan Institutional Animal Care and Use Committee (#PRO00006234, #PRO00008306) and collections made through permits from the Servicio Nacional Forestal y de Fauna Silvestre (029‐2016‐SERFOR‐DGGSPFFS, 405‐2016‐SERFOR‐DGGSPFFS, 116‐2017‐SERFOR‐DGGSPFFS) and Ministerio del Ambiente y los Recursos Naturales de la República de Nicaragua (DGB‐IC‐058‐2017, DGPNB‐IC‐019‐2018, DGPNB‐IC‐020‐2018, DGPNB‐IC‐002‐2019).

**TABLE 1:** Collection, staining, and scanning information for 23 museum specimens used in this study

Clade	Taxon	Museum	Specimen	SVL (mm)	Mass (g)	Head diameter (mm)	Number of scans	Days stained	Diffusion rate (mm/day)	Preservation age (years)
Aniliidae	*Anilius scytale*	UMMZ	248356	495	14.27	7.14	4	4	0.893	2.98
Colubrinae	*Chironius fuscus*	UMMZ	245047	708	90	10.77	4	5	1.08	3.93
	*Psuestes sulphureus*	MUSM	37565	1,840	1,250	30.43	2	12	1.27	2.09
	*Lampropeltis abnorma*	UMMZ	247095	247	91.4	11.16	4	6	0.93	1.32
	*Leptophis ahuetulla*	MUSM	37345	565	27.66	9.12	2	5	0.91	1.95
	*Tantilla melanocephala*	UMMZ	246845	255	7.39	5.51	4	3	0.92	1.95
Dipsadinae	*Imantodes cenchoa*	UMMZ	246810	876	30.76	7.93	4	5	0.793	2.67
	*Helicops angulatus*	UMMZ	246805	427	63	12.95	4	5.5	1.18	2.45
	*Helicops leopardinus*	UMMZ	246808	685	220	18.72	4	9	1.04	2.90
	*Leptodeira septentrionaius*	UMMZ	247099	654	113.2	14.85	4	6	1.24	1.37
	*Nothopsis rugosus*	UMMZ	248404	257	6.41	5.45	4	4	0.68	1.33
	*Oxyrhopus melanogenys*	MUSM	37417	230	10.77	6.41	4	6	0.53	3.47
	*Xenopholis scalaris*	UMMZ	246854	271	7.61	5.94	3	4	0.74	1.81
Elapidae	*Micrurus lemniscatus*	MUSM	35905	725	50	9.31	2	4	1.16	2.61
	*Micrurus nigrocinctus*	UMMZ	247142	717	64.8	12.56	4	6	1.05	1.69
	*Micrurus obscurus*	UMMZ	246859	261	5.19	6.34	2	5	0.63	2.38
	*Micrurus surinamensis*	MUSM	37353	421	32.47	10.02	4	7	0.72	3.16
Lamprophiidae	Aparallactus capensis*	UMMZ	61599A	104	8.4	3.26	4	3	0.54	95.62
	Atractaspis bibronii*	UMMZ	209986	340	16	6.61	4	4	0.83	25.58
Viperidae	*Bothrops bilineatus*	UMMZ	245084	744	85	15.93	4	5	1.59	3.62
	Causus rhombeatus*	UMMZ	65828	410	58	14.55	4	8	0.91	91.62
	*Lachesis muta*	UMMZ	248369	763	145	21.2	4	11	0.96	3.53
	*Porthidium nasutum*	UMMZ	247139	297	19.1	12.74	4	4	1.59	1.66

Abbreviations: MUSM, Museo de Historia Natural de la Universidad Nacional Mayor de San Marcos, Lima, Peru. Asterisks (*) denote historic specimens; SVL, snout‐vent length; UMMZ, University of Michigan Museum of Zoology, USA.

### 2.2 Workflow for staining and micro‐CT scanning

#### 2.2.1 Scheduling scans

For 18 specimens, we conducted two unstained and two stained scans per specimen: (a) skeletal scan of the entire specimen prior to staining, (b) skull scan of the head as a region of interest (ROI), (c) diceCT scan of the entire specimen, and (d) diceCT scan of the head ROI. The remaining five specimens were scanned only twice: a skeletal (a) and diceCT (b) scan of the entire specimen for *Pseustes sulphureus*, and a skull ROI (c) and diceCT ROI (d) each for *Leptophis ahaetulla, Xenopholis scalaris, Micrurus lemniscatus,* and *Micrurus obscurus* (see Table 1). These specimens were stained and scanned early in the development of our methodology and were included in the study because they demonstrate inadequate packing/staining and/or broaden the range of body sizes.

Scan times were ~14 min for each skeletal scan and ~3.75 hr for diceCT at their respective standard parameters (Table S1). Entire body and ROI diceCT scans were performed sequentially overnight using a batch scan program. Given the significantly longer scan time of diceCT compared to skeletal scans, scanning at night maximized workflow efficiency and data generation during the day, and allowed the specimen to settle in the packing media. Overnight batch scanning was paramount to ensuring a high‐throughput workflow pace; it also allotted extra time for any unexpected delays and setbacks we experienced.

#### 2.2.2 Iodine staining

Once skeletal scans were complete, we stained all specimens by submersion in 1.25% (total solute) Lugol's iodine solution (I_2_ + KI + H_2_O) in the dark, following Gignac and Kley (2014). Preparation protocols for reagents and solutions are provided in Appendix S1. We prepared approximately 3.85 L of Lugol's iodine solution at a time. To ensure specimen quality and longevity, we only stained preserved specimens once, although it is unknown what consequences, if any, arise from multiple bouts of staining. Given that optimal staining duration varied per specimen, we planned diceCT scans at least 1–2 weeks in advance.

**FIGURE 1:**
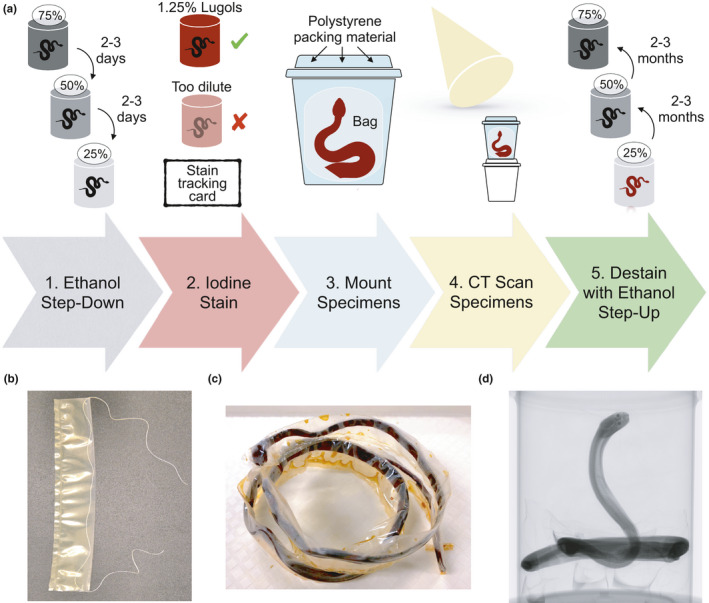
Flow chart of the diceCT process. (a) Illustrated representation of the five steps between selecting a preserved specimen in 75% ethanol and returning it fully destained back to the collection. Photographs (b), (c), and (d) are the critical components of packing a diceCT snake specimen. (b) Partially heat‐sealed bag with an encased string to facilitate specimen positioning within the bag, plus a staining specimen card to keep track of staining progress, as described in Section 2.2.3; (c) Stained specimen that has been pulled through the plastic bag using the string (see Section 2.2.3) and fully heat sealed to prevent desiccation during the scan; (d) A packed, stained specimen in the CT scanner. The packing medium is foam packing peanuts (30% recycled polystyrene, Uline, WI, USA) purposefully chosen for their low density, making them not visible in the scan. This mounting position with an elevated, isolated head is ideal as it allows for optimal resolution on cranial scans (see Section 2.2.4). We only used ethanol destaining in this study, but low concentrations of sodium thiosulfate can be used to accelerate destaining (see Section 4.1)

**FIGURE 2:**
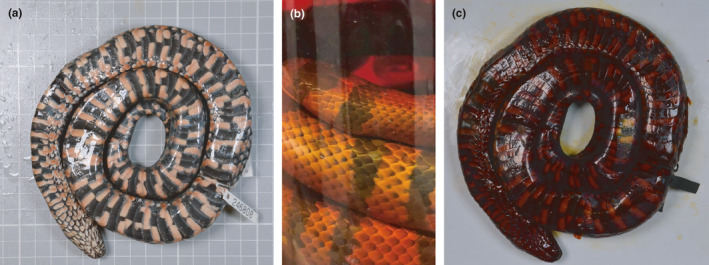
Visual indicators of successful and incomplete iodine staining in preserved snakes. (a) Ventral view of an unstained snake specimen. (b) A specimen immersed in 1.25% Lugol's iodine, which has become partially transparent. The transparent solution indicates incomplete saturation of the specimen and should be replaced with freshly made 1.25% Lugol's iodine. (c) Ventral view of the same specimen shown in (a), fully stained. Note the dark amber colouration and obscuring of body patterns. Specimen in (a) and (b) is a *Helicops leopardinus* (UMMZ 246808) stained for 9 days in 1.25% Lugol's iodine solution. Specimen in (c) is an actively staining *Lampropeltis abnorma* (UMMZ 247095)

Specimens were downgraded in stepwise concentrations of EtOH (75%, 50%, 25%); spending 2–4 days at each concentration (Figure 1, Step 1). The EtOH downgrade may lessen the effects of osmotic shock of moving specimens from alcohol to the water‐based Lugol's iodine solution, and vice versa (pers. obs. S. Callahan, GE Schneider; Simmons, 2014). Specimens were then immersed in large containers of 1.25% Lugol's iodine (Figure 1, Step 2). To assess whether the 1.25% Lugol's iodine had completely perfused the submerged specimen, we examined the opacity of the solution every 24 hr (Figure 1, Step 3). Complete tissue saturation was indicated, in part, when the solution was opaque for at least 72 hr (Figure 2). If the solution changed from opaque to translucent, this indicated incomplete diffusion and the solution was replaced with fresh 1.25% Lugol's iodine and again monitored for saturation. The skin of adequately stained specimens was dark amber in color, which often obscured any external color patterns on the specimen that were visible prior to staining (see Figure 2 for ideal staining). Specimens with incomplete diffusion typically looked “under‐stained,” that is, skin was a light red or yellow in external appearance.

**FIGURE 3:**
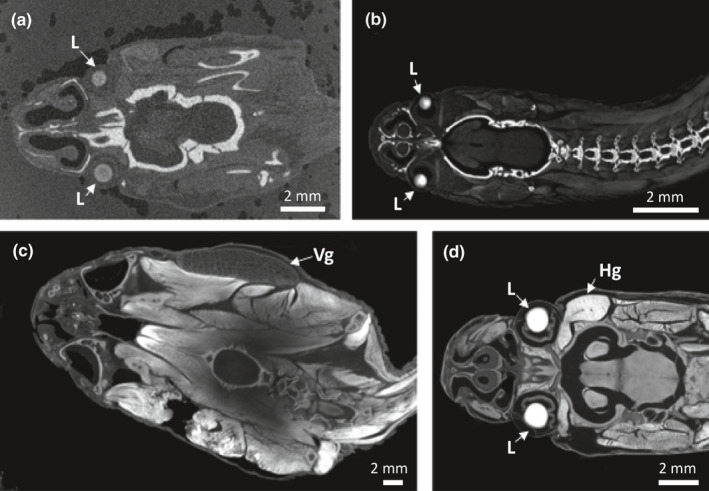
Examples of variation in staining quality among snake head region of interest. (a) Under‐stained specimen that is also distorted by inappropriate foam packing material (2 inch soft foam sheets, Uline, WI, USA). (b) Understained specimen that is well‐contrasted with packing peanuts as packing material. Note the high contrast (oversaturation) of the skeletal system and low contrast of soft tissues. (c) Moderately understained specimen packed in packing peanuts (30% recycled polystyrene, Uline, WI, USA). Note that the left venom gland was dissected before preservation. (d) Well‐stained specimen, with an overstained Harderian gland, packed in packing peanuts. The left Harderian gland was dissected before preservation. Specimen (a) is *Xenopholis scalaris* (UMMZ 246854), (b) *Aparallactus capensis* (UMMZ 61599), (c) *Lachesis muta* (UMMZ 248369), and (d) *Oxyrhopus melanogenys* (MUSM 37417). Specimens were stained in 1.25% Lugol's iodine. L = Lens, Hg = Harderian gland, Vg = Venom Gland [Correction added on 15 July 2021, after first online publication: Figure 3 caption has been corrected in this version.]

If optimal staining duration could not be determined by inspecting solution opacity and/or external appearance of specimens, we performed a quality assessment scan to assess the staining progress (Figure 3). A brief scan was conducted at the standard diceCT parameters (see Table S1) and aborted a few minutes after the scan began, as we only needed a few tomographic slices to assess soft‐tissue contrast. If the specimen was under‐stained, there was a visible diffusion gradient (Figure 3). If the specimens was overstained, there was very minimal contrast among the internal soft tissues.

We also tested for the potential effects of specimen size on staining duration. We took standard measurements of specimen size (SVL, mass, and head diameter) for 20 specimens preserved recently (1–3 years old), and three historical specimens (25–95 years old) already present in the UMMZ collections (Table 1). Effects of specimen mass were only tested for individuals that were weighed prior to preservation (*n* = 20) to minimize measurement error due to preservation fluid. We calculated diffusion rate by dividing the radius (mm) of the head by total staining duration (d).

#### 2.2.3 Packing

Any movement during scanning will create a misalignment of the center of rotation, yielding poor or unusable data (e.g., blurred edges within two‐dimensional [2D] tomography slices). To ensure high‐quality data, specimens should be packed to adequately restrict specimens to prevent movement during scanning.

Snake specimens are typically fixed in a tightly coiled spiral during the preservation process to accommodate their elongate, limbless bodies in the specimen jars. This presents unique challenges for packing snakes for CT scanning. Limbed vertebrate specimens are typically preserved in a manner that separates the limbs from the rest of the body, and they can be prepared for scanning by packing them into a flat and rectangular bag, without excessive manipulation of the specimen itself. In turn, head ROI scans of limbed vertebrates are relatively simple to conduct without interference from other anatomical structures. The coiled position of preserved snakes is adequate, although not ideal, for full‐body scans, but it becomes problematic for head ROI scans because the head is not spatially separated from the body coils. As a result, the X‐rays will attenuate as they are absorbed through or deflected off non‐ROI parts of the body. This problem is more pronounced for ROI scans because the head is often nested between large body coils, and the resulting scans of the head ROI are reduced in quality. Additionally, coiling the specimen upon itself leaves a considerable amount of air trapped in the packing bag, which increases the potential for desiccation.

To address these challenges, we prepared coiled specimens for scanning by using customized plastic bags that had been cut and heat sealed. We cut poly tubing plastic (Uline, WI, USA) to 5–10 cm longer than the total length of each snake and sealed lengthwise, leaving the ends unsealed (i.e., open) (Figure 1b). We also placed a piece of string, twice the length of the plastic bag, inside the bag with excess string coming out of the open ends. One end of the string was tied around the specimen's neck, and then the specimen was pulled through the bag by pulling the loose string on the other end. The string was removed and the anterior‐end of the bag was heat sealed, leaving some extra space at both ends of the specimen. Any metal tags were replaced with paper tags until after destaining was complete.

To keep specimens in place during scanning, we packed them into appropriately sized containers. The container should be large enough to manipulate the specimen easily and tightly pack the specimen with minimal packing media. We typically chose wide mouthed, round containers (5–15 cm diameter; Uline, WI, USA). We found “anti‐static packing peanuts” (30% recycled polystyrene, Uline, WI, USA) to be the ideal packing media because the X‐rays fully penetrated the packing peanuts and produced minimal noise when rendering the data (especially compared to larger foam sheets, see Figure 3a). They are also easy to source, reusable, and inexpensive. We tightly filled the empty spaces around the positioned specimen with peanuts to hold the specimen in place during the scan.

We positioned specimens in an ascending spiral with the neck and head separated by strategic layers of packing media, with the head in the middle of the container pointing upwards (Figure 1d). Once the container lid was sealed and given a specimen tracker tag (Figure 1, Step 2), it was left to settle to minimize the risk of the specimen moving during scanning. Specimens were left for a minimum of 30 min for skeletal scans and 2 hr for diceCT scans. We performed full body and head ROI scans sequentially to prevent the need for repacking of specimens between scans.

#### 2.2.4 Mounting

After the specimen had settled in its packing container, we placed it on top of a similarly sized or larger mounting container (Figure 1, Step 3). Mounting containers are empty containers that create spatial separation between the metal platform and the specimen. We placed the stacked containers in the middle of the scanner platform and manipulated using the zoom and y‐direction platform joysticks and/or by manually moving the stacked containers. Platform manipulation in the x‐direction on the scanner was locked in all scans. We manually repositioned the stacked containers at various degrees of rotation to ensure the ROI always remained visible to the detector panel.

#### 2.2.5 Scanning parameters

We conducted all scans on a Nikon Metrology XTH 225ST μ‐CT scanner (Xteck, Tring, UK). We conducted skeletal scans at 85 kilovolts (kV, voltage), 200 microamperes (uA, amperage), 250 millisecond exposures (ms), 1601 projections, with 2x‐frame averaging. We conducted diceCT scans at 85 kV, 200 uA, 250 ms, 3,141 projections, with 16x‐frame averaging (Table S1). Scans where the voxel size was less than the power were conducted at 120uA. We reconstructed raw tomography projections using CT‐3D Pro (Nikon Metrology, Tring, UK), which generated approximately 2000 cross‐sectional, which generated approximately 2000 cross‐sectional images in tagged image file format (TIFF) per dataset. For visualization, we imported the reconstructed images into Volume Graphics (VG) Studio Max version 3.3 (2019, Volume Graphics, Heidelberg, Germany) where they were compiled into 3D renders for segmentation and anatomical analysis.

#### 2.2.6 Destaining

After the diceCT scanning was completed, we destained specimens with a series of EtOH solutions (25%, 50%, and 75%), leaving the specimen in each EtOH concentration for 2–3 months (Figure 1, Step 5). We periodically replaced the EtOH solution when it reached near‐complete iodine saturation, as indicated by the dark amber color of the liquid.

### 2.3 Postscanning data management and analysis

#### 2.3.1 Data storage and access

All scans produced from this study are available on Morphosource (see Table S2). Once scans were complete, the tomographic projections (.tiffs) were reconstructed into a dataset comprising a cross‐sectional image slice stack (each image is a single orthogonal slice through the specimen). Each cross‐sectional image slice stack was exported as a 16‐bit tiff stack. In addition to the image stack, a Volume Graphics project file (.VGL) was created. This project file facilitates the opening and viewing of the data in Volume Graphics 3.2. The final dataset for each specimen included: raw tomographic projects (.tiffs), cross‐sectional image stack (.tiffs), and a Volume graphics project (.VGL). The dataset for each specimen was then backed up onto a pair of 5TB external hard drive (one primary and one backup).

#### 2.3.2 Digital segmentation of hard and soft tissues

We conducted segmentation in VG Studio Max v3.2, aided by the use of a Wacom Cintiq 22HD tablet version 6.37–3 (Wacom Co., Ltd., Kazo, Saitama, Japan). We used a combination of the “draw” and “region‐growing” tools to segment bone from skeletal scans and soft‐tissue anatomy from diceCT scans. We identified the range of grayscale values (GV) of the anatomical structure of interest using the “navigation cursor” tool, which were used to constrain the selection made by the draw tool. For the region growing tool, a single pixel or cluster of pixels was selected by the user and a gray value threshold set as the ± range of pixels that will be included in the selection. This pixel range varied among specimens but the typical threshold value was ±1,000 within the gray values of the anatomical structure of interest.

## 3 RESULTS

We stained and scanned a total of 23 specimens in 31 weeks (Table 1), generating 41 skeletal scans and 41 diceCT scans (82 scans in total) with 18 specimens consisting of both full body and ROI head scans (mean = 2, range = 0–2 per week). DiceCT scans of the head ROI had higher resolutions (range 0.01001–0.02923 voxels) than the full body diceCT (range 0.05116–0.08475 voxels) due to constraints in packing coiled specimens and sequential scan setups (Table S1). Nevertheless, both head and full body diceCT scans yielded good quality data for the variety of downstream applications we detail below.

**FIGURE 4:**
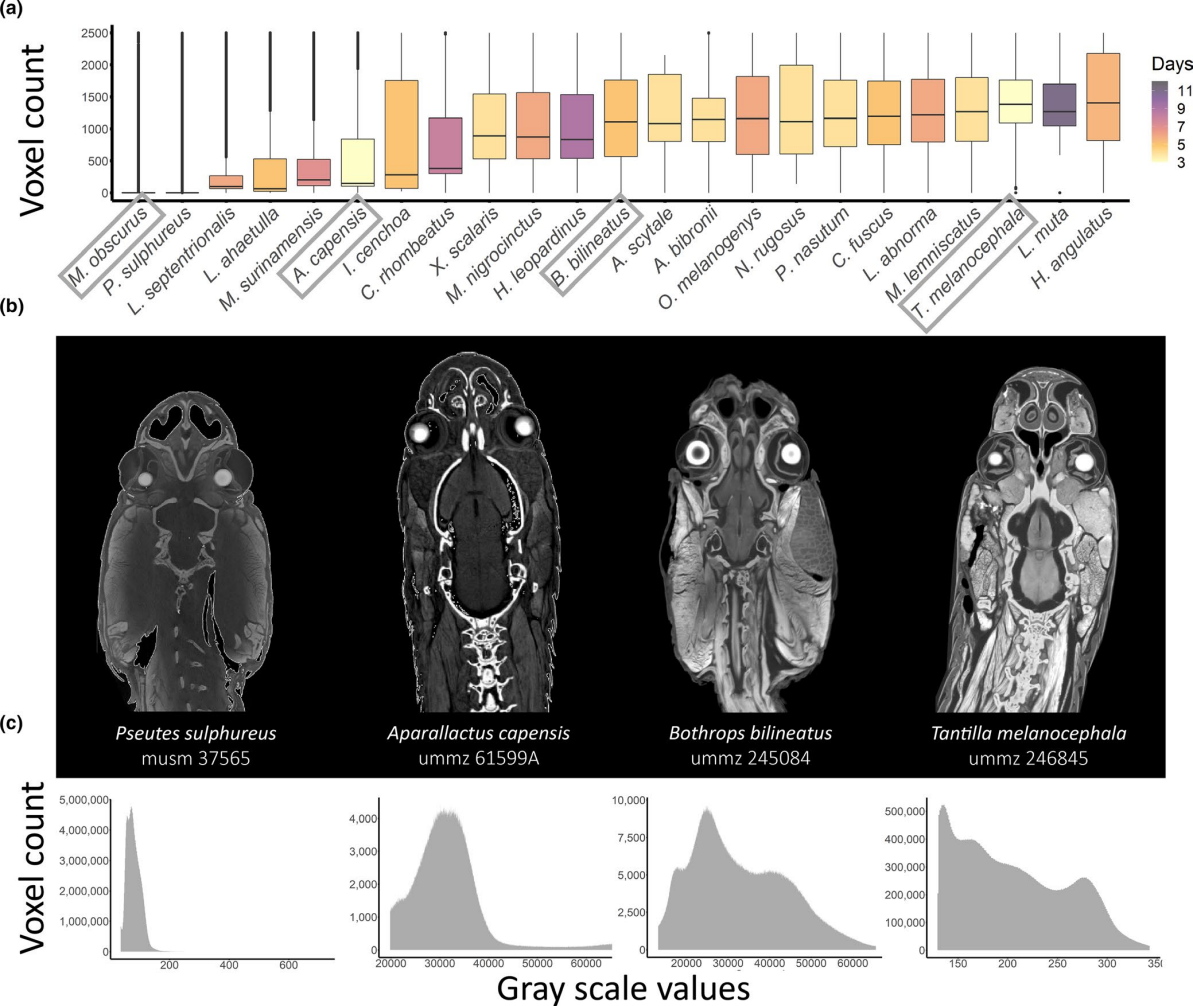
(a) Histograms showing mean and range of grayscale values (GV), colors represent total duration in 1.25% Lugol's iodine solution (days), gray boxes indicate select specimens in (b‐c); (b) dorsal tomography slices of snake heads; (c) corresponding histograms show distribution of GV for select specimens. Note the variable axes on histograms

**FIGURE 5:**
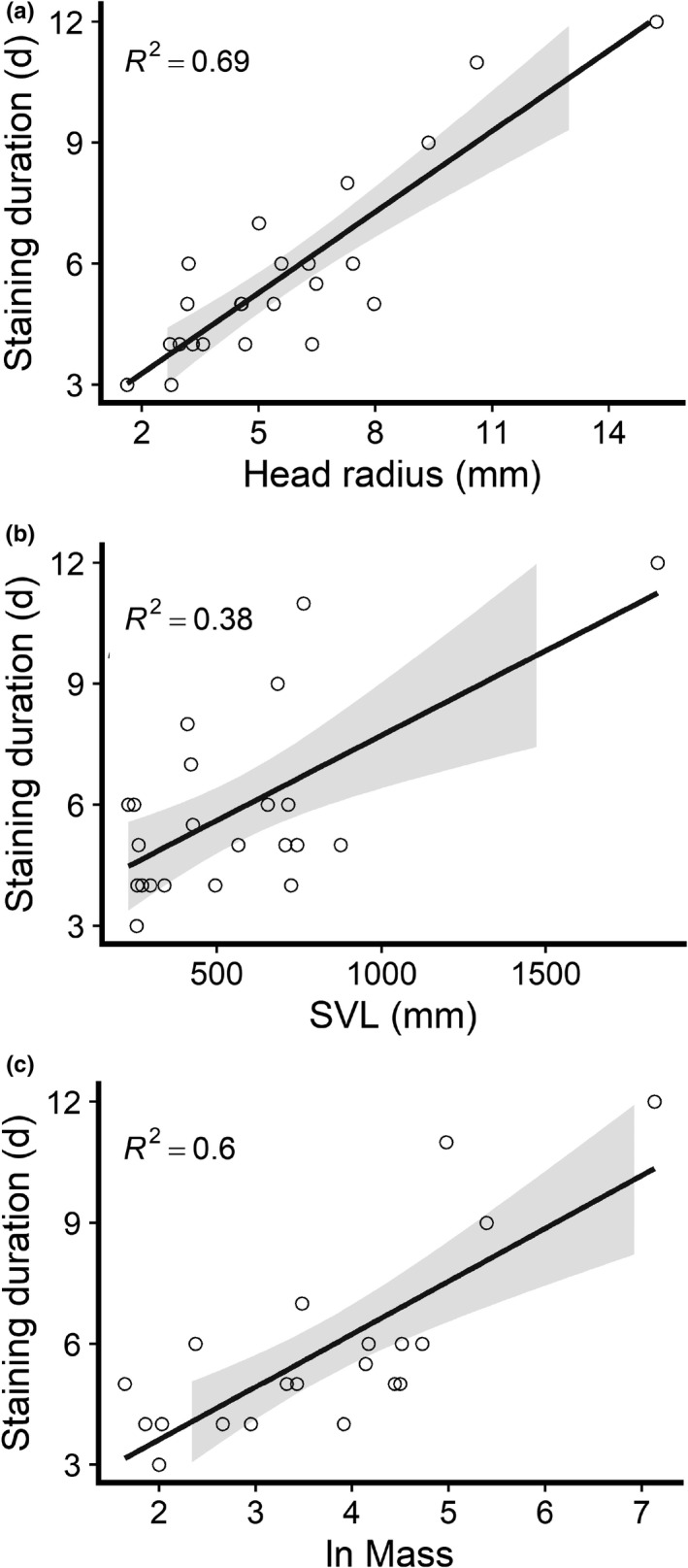
The relationship between specimen size and duration in 1.25% Lugol's iodine solution: (a) snout‐vent length (SVL), (b) mass, and (c) head radius. Radii were calculated from the diameter taken at the widest point. 95% confidence intervals shown in gray. Note the ln log scale for mass. Data for SVL and head radius are from 23 species (*n* = 23 individuals) and data for mass are from 20 species (*n* = 20 individuals) from the snake families Aniliidae, Dipsadinae, Colubrinae, Elapidae, Lamprophiidae, and Viperidae

Optimally stained specimens resulted in 2D tomography slices with consistent contrast among all tissues. Under‐stained specimens generated scans with a narrow GV range, overstained specimens corresponded to broad GV range with overall low voxel counts across values, and optimally stained specimens had a relatively narrower GV range but consistently higher voxel counts across those values (Figure 4). GV is a way of visualizing x‐ray attenuation (i.e., a localized reduction in X‐ray intensity). On a GV histogram, multiple peaks and a broad range of GV correspond to optimal contrast, and narrow range (single peaks) tends to correspond to lower contrast (Figure 4). However, these qualitative assessments of optimal contrast were based on subjectivity of the user viewing the scan and may change depending on which soft‐tissue structures the user is most interested in. The most common effect of prolonged staining was an uneven uptake of the iodine solution for some tissues over others, yielding a narrow GV range with overall values that near, match, or exceed the GV limits of the UMMZ Nikon XTH 225S µ‐CT scanner. Optimal scans and GV ranges were not directly associated with the total staining duration of specimens (Figure 4a), as there is an interaction with body size. To test the potential effects of specimen size on staining duration, a linear regression analysis was performed on natural log‐transformed data. We found that the radius of the head was significantly correlated with the number of days specimens were in 1.25% Lugol's iodine (Figure 5a, *F*_1, 21_ = 47.70, *p* <.001). There was also a weaker correlation between size (both SVL and mass) and the number of days specimens were in 1.25% Lugol's iodine (Figure 5b and c, SVL *F*_1, 21_ = 12.96, *p* <.01; mass *F*_1, 18_ = 27.44 *p* <.001). There was no correlation between specimen age and number of days specimens were in 1.25% Lugol's iodine (*F*_1, 21_ = 0.15, *p* =.7073). The mean iodine diffusion rate was 1 mm per day (*SD* = 0.34 mm).

**FIGURE 6:**
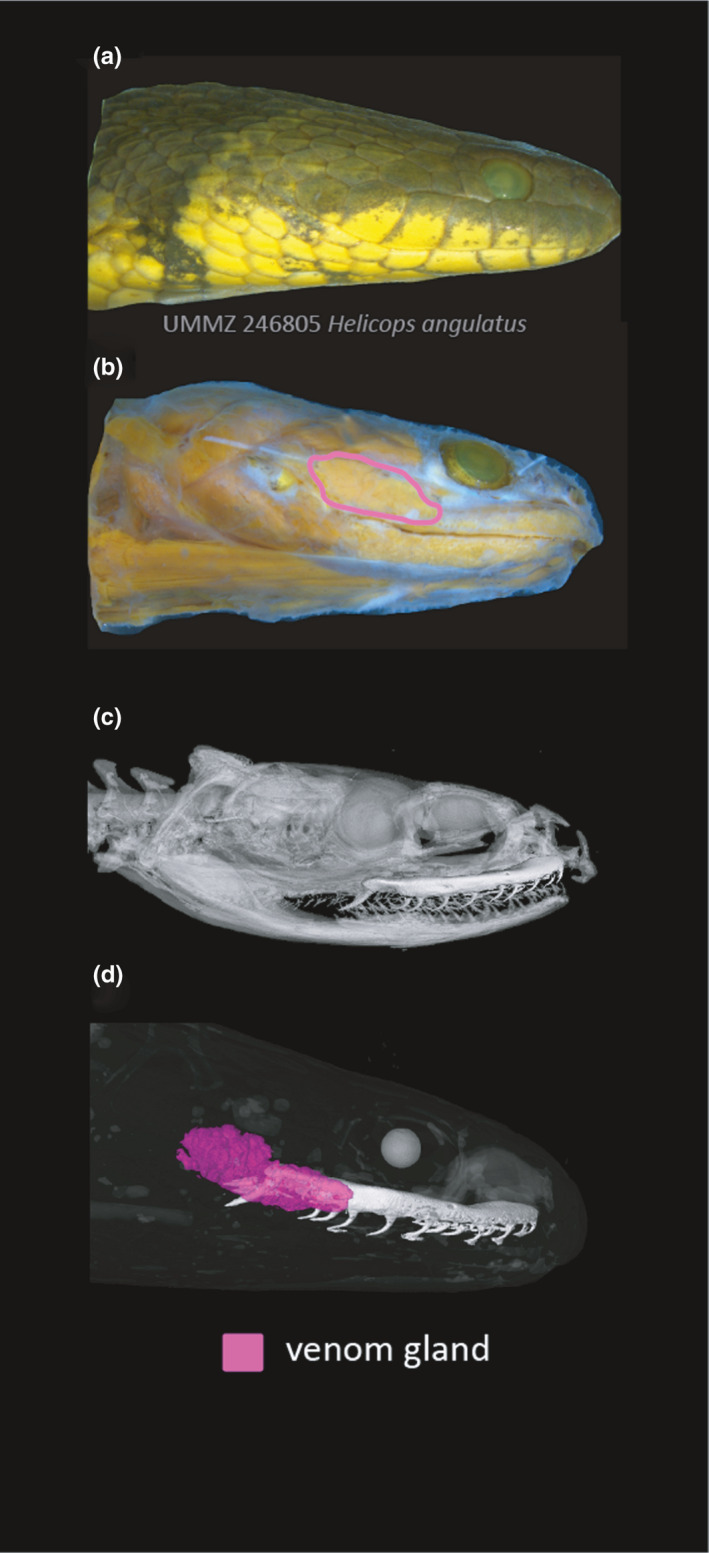
Integrating physical dissections with skeletal and diceCT scans can help resolve complex and/or highly variable anatomy. Lateral view of the same preserved specimen: (a) undissected, (b) skinned with venom (Duvernoy's) gland highlighted, (c) skeletal 3D render with the maxillary bone segmented, and (d) diceCT 3D render of the venom gland segmentation and maxillary bone. Eyes are rendered in white for positional reference. Specimen is *Helicops angulatus* (UMMZ 246805) [Correction added on 15 July 2021, after first online publication: Figure 6 has been corrected in this version.]

**FIGURE 7:**
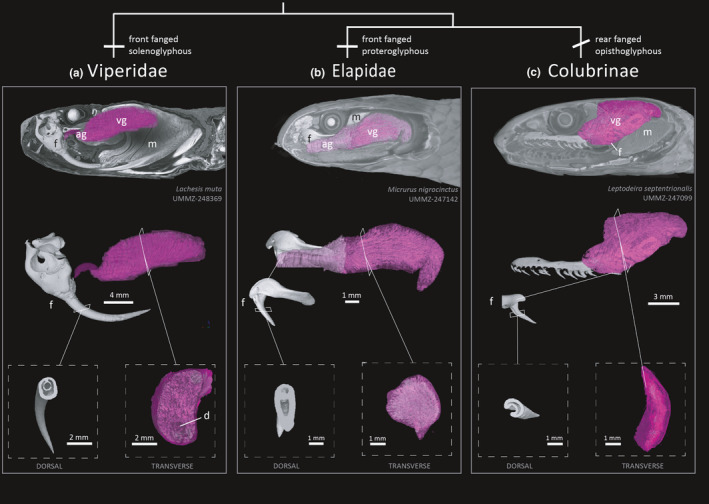
Combining skeletal and diceCT datasets to explore morphology in venom delivery systems in snakes. Fang morphology and positioning on the maxilla bone differs between (a) Viperidae, tubular front fangs (solenoglyphous), (b) Elapidae, hollow front fangs (proteroglyphous), and (c) Colubridae, grooved or unmodified rear fangs (opisthoglyphous). DiceCT can be used to vizualise and quantify soft‐tissue anatomy (venom and accessory glands, duct connections, muscle) with fang traits to build an integrative comparison of venom systems across taxa. ag = accessory gland, d = duct, f = fang, m = muscle. vg = venom gland

**FIGURE 8:**
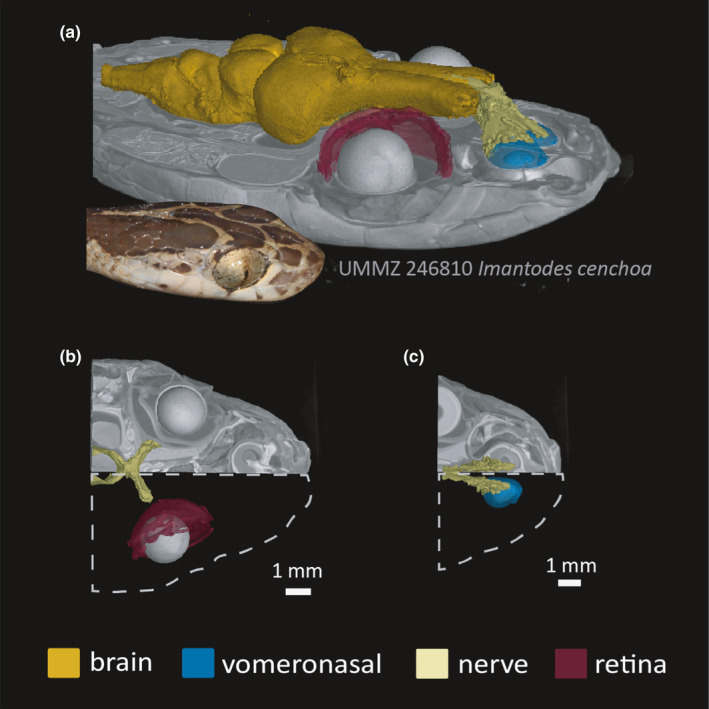
DiceCT data allows for morphological comparisons in situ, which makes it an important technique for studies of trait evolution, especially systems that evolve in unison such as neural and sensory anatomy. (a) Dorso‐lateral view of a whole‐brain segmentation of *Imantodes cenchoa* (UMMZ‐346810). (b) Dorsal view of a tomography slice with 3D segmentations of the visual system. (c) Dorsal view of a tomography slice with 3D segmentations of the vomeronasal system. Image credit: Consuelo Alarcón Rodriguez

**FIGURE 9:**
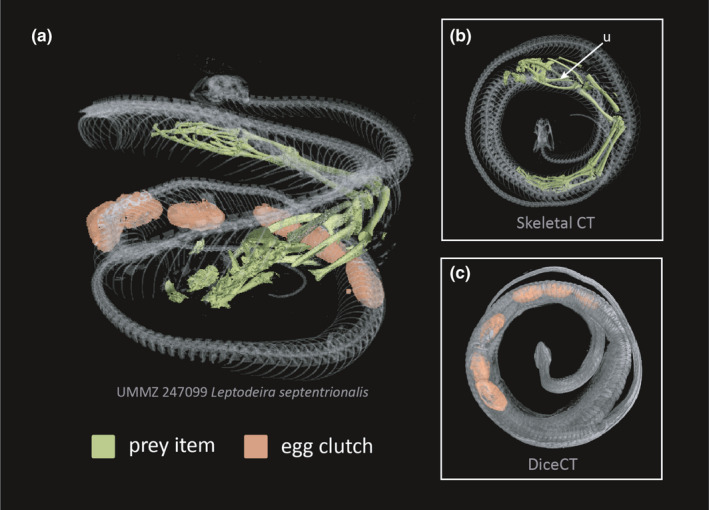
Natural history bycatch: two full‐body scans of the same specimen (UMMZ 247099) show a recent prey item and gravidity in a female *Leptodeira septentrionalis*. (a) Lateral view of combined dice and skeletal CT scans. (b) Ventral view of snake skeletal scan with prey segmentation in green. Anuran prey was identified by presence of the urostyle (u). (c) Ventral view of snake diceCT scan with eggs segmentation in orange

Prior to scanning, the majority of specimens had small, unilateral dissections to remove tissue from one side of the specimen for use in ongoing molecular projects. Iodine uptake at areas of dissection was considerably quicker than low‐density structures such as the epidermis or stomach, resulting in oversaturation of tissues adjacent to dissection sites (e.g., cephalic glands). On 2D tomographic slices, these overstained structures appeared oversaturated (i.e., very bright and higher GV), which lowered the contrast of surrounding soft tissues, and subsequently shifted GV ranges across the entire specimen, which resulted in lower contrast even among adequately stained soft tissues. This effect was especially problematic for visualizing small and/or discrete soft‐tissue anatomies, such as nerves and unmyelinated encephalic structures, that failed to render (i.e., invisible) or appeared undifferentiated from surrounding structures. Additionally, external tissues with high surface‐to‐volume ratio (e.g., tongue and epidermis) were often oversaturated in under‐stained specimens. Deeper internal tissues (e.g., glands, muscle, bones, and neural tissue) had little to no iodine uptake in under‐stained specimens, and they showed limited ultrastructural morphology and tissue differentiation in appearance when viewed in 2D tomography slices (Figure 4b, *Pseutes sulphureus*). Despite the variability in staining quality, we successfully segmented many internal features from most scans, including venom delivery systems (Figures 6, 7), neurosensory structures (Figure 8), and diet items and developing eggs (Figure 9).

We used an EtOH destaining protocol without the use of additional solvents (e.g., sodium thiosulfate), which resulted in highly variable destaining duration depending on specimen size. Smaller specimens (e.g., *Aparallactus capensis*; 104 mm SVL) were adequately destained after 2 months; larger specimens (e.g., *Psuetes sulphureus*; 1,840 mm SVL) took over a year to fully destain. Some specimens initially displayed altered morphological characteristics from the staining process, especially external and internal discoloration of soft tissue and dehydration. The effects of specimen dehydration were particularly visible in the eyes, which presented with concave and wrinkled corneas. However, we found that discolouration and dehydration were fully reversible over time using the EtOH downgrading and upgrading method outlined (Figure 1 Step 1 and 5; Figure 2).

## 4 DISCUSSION

We present a protocol to efficiently stain, pack, mount, scan, and destain museum specimens from a taxonomically diverse range of snakes, applicable to high‐throughput data generation for any elongate vertebrate. Our protocol optimizes quality of µCT data and 3D reconstructions, maximizing usability and longevity of “digital specimens” without compromising the integrity of physical museum specimens. There are many benefits of incorporating diceCT scanning into µCT workflows, as it creates a near‐complete digital copy of internal anatomy that can be shared widely with limited destruction to specimens (cf. to traditionally dissection methods). However, challenges for diceCT include a substantial time commitment in the staining and destaining process, complex analyses of 3D soft‐tissue anatomy, and the potential risk of long‐term damage to specimens, especially if specimens are stained more than once. Here, we recommend best practices for optimizing µCT workflows for snakes while mitigating potential risks, and we discuss the potential role for high‐throughput generation of diceCT data in research within ecology and evolution.

### 4.1 Packing snakes for µCT scanning

We found that creating form‐fitted customized bags provided several advantages for packing coiled snakes. Foremost, this enclosing bag allows for unrestricted positioning of the specimen, which is especially ideal for packing a specimen for ROI scans. The bag also reduces the amount of trapped air, which can dehydrate specimens. Excess iodine solution sometimes collected in the bag, which ultimately caused noise during scanning; vacuum sealing mitigated this issue but increased the potential for skin deformation through contact with the bag. Positioning the snake in a loose ascending spiral, with separation of the head and neck, allowed for minimal attenuation otherwise caused by interference from surrounding structures (Figure 1). The ideal packing position for snakes would be an airtight bag, with the specimen stretched out entirely straight; the scan quality of this specimen could be maximized if scanned helically. However, with the UMMZ scanner, and many types of scanners, helical scanning is currently not an option, and stretching most snakes out their entire length would be too long for the detector panel and or significantly reduce resolution. We recommend that if specimens are being collected for the express purpose of diceCT scanning, then they should be preserved flat with as few spirals as is practical for storage (Figure S1), but note there are new resources for “unwinding” specimens postscanning (e.g., Williams et al., 2020). These protocols for packing snakes can be applied to other elongate vertebrates including fishes (e.g., hagfish, lampreys, and eels), amphibians (caecilians, sirenid, and amphiumid salamanders), amphisbaenians, and legless lizards.

### 4.2 Effects of staining on specimens

We did not explicitly test how the effects of specimen age, preservation, and storage affected the quality of diceCT data. Most specimens used in this study were collected recently (2016–2019), immediately preserved and stored with knowledge that they would ultimately be diceCT scanned. We found that specimen age and duration of preservation were not correlated with total duration of staining, and the three older specimens (collected circa 1950s; Table 1) used in this study did not present any noticeable deviations in staining and or scan quality. Nevertheless, other studies have shown that diceCT of older specimens (e.g., stored in 70% EtOH > 70 years) yield 2D tomography slices with narrow GV ranges and thus poorly differentiated soft‐tissue anatomy (Gignac et al., 2016; Hughes et al., 2016). Future studies should aim to test the effect of specimen age as well as how preservation and storage affect quality of diceCT scans.

The physical effects of the 1.25% Lugol's iodine appeared to be fully reversible using an EtOH destaining protocol. This protocol was selected over other existing destaining methods in the interest of maintaining specimen quality and longevity. Using a <10% sodium thiosulfate solution for iodine destaining can dramatically reduce the staining duration and immediately revert specimens to their original color (Schmidbaur et al., 2015). However, preliminary evidence suggests that using a sodium thiosulfate solution increases calcium solubility that potentially causes decalcification of ossified structures (Mataic & Bastani, 2006). Thus, we took a cautious approach and chose only EtOH destaining, which resulted in substantially greater destaining duration, particularly for large specimens (up to 1 year). The sodium thiosulfate method is used regularly and successfully in other laboratories with no detectable negative effects, provided that the concentration of sodium thiosulfate is kept very low (<1%), and the specimen remains in sodium thiosulfate for short periods of time (pers obs, J.A.G.). Demineralization has also been observed in avian specimens that were immersed in 1.25%–3.75% Lugol's iodine for longer durations, that is, 5–10 weeks cf. 3–12 days used in the present study (Early et al., 2020). Our staining durations are quicker than those reported in other studies (Gignac et al., 2016), which may be due to variations in our protocol (e.g., EtOH downgrade, size of staining vessels), laboratory set up (e.g., ambient temperature) and/or the high surface‐to‐volume ratio of snakes. More studies are needed on a variety of taxa to test the potential effects of staining and destaining on museum specimens; we view our approach as conservative but successful for minimizing the known effects of iodine staining to specimens.

### 4.3 Financial and temporal considerations

High‐throughput diceCT projects require a sizable amount of financial and temporal commitments, in addition to a number of key personnel. Researchers need access to a µCT scanner for prolonged and uninterrupted scanning, which we mainly performed overnight. These scanning sessions must be planned in advance to ensure that specimens are removed from the staining solution at the appropriate times, which can be challenging because specimens of varying sizes stain at different rates (Figure 5). In addition to reserving µCT scanners for prolonged times, researchers should anticipate delays for setbacks and maintenance of CT scanner equipment. During this study, our timeline was frequently altered/extended due to necessary but unscheduled maintenance, timing filament changes, and unexpected program errors, which resulted in the subsequent abortion of batch scan programs.

An estimate of financial costs associated with diceCT scanning at the UMMZ is provided in Table 2. Based on these estimates, our approximate cost of generating a single diceCT scan of a snake was $216 (approximately 4.5 hr to scan at $48/h), which we present as an exemplar price point to initiate budget discussions for researchers considering a diceCT project. This estimate is based on hourly operational costs at a facility that is already set up for diceCT scanning (Table 2). However, these costs will vary considerably depending on workstation requirements, type of CT scanner, how time is billed for shared CT scanners, and number of technicians/personnel needed for scanning. Costs could be substantially lowered by sharing scanners, software, and training with other research/medical laboratories. A variety of open‐access and free‐to‐use software are available for analysis and segmentation of CT data including Dragonfly, MeshLab, 3D Slicer, FIJI, and Blender. Choice of software for rendering and segmenting scans depends on the intersection of many factors including cost, computing power, and available time to users to learn software (for discussion see Buser et al., 2020). Finally, a data management plan is vital to ensure data longevity, access, and dissemination for research and educational initiatives (see Appendix S2 for details of the data management plan used in this study).

**TABLE 2:** Operational costs to set up diceCT scanning facilities and estimate of since diceCT scan after set up. Estimates based on costs at University of Michigan CT facilities

Item	Estimate (USD)	Description/model used at the UMMZ
Computer workstation	$6,500–$10,600	RMC 1040: HP Z4 G4, Intel Core i9, 3.3–4.1 GHZ, 16 Mb Cache, 8 × 16 GB RAM (128 total), Nvidia Quadro RTX 5000 (16 GB RAM). Hewlett‐Packard (CA, USA)
Touchscreen monitor	$1,000–$2,000	Optional, for segmentation. Wacom 21” Cintiq 22HD. Wacom (Japan)
Data storage: External hard drives	$114.99 per drive	5TB external hard drive, Seagate (CA, USA)
Cloud storage	Amazon Cloud: $59.99/Tb	Prices reflect yearly subscriptions, which vary by vendor
	Dropbox: $99.99/Tb	
	Google Drive: $99.99/Tb	
RAID storage	$150–$460	Price varies by vendor. Estimates from Western Digital (CA, USA)
Volume Graphics Studio Max	$12,000, plus $2,100 per year service contract	Volume Graphics Ltd. (SC, USA)
ORS Dragonfly	Free (academic license)	Segmentation software. ORS (QC, Canada)
Nikon XTH 225ST	$600,000–800,000	Only if buying a CT scanner.
		Micro‐CT scanner, Nikon (Japan)
Nikon XTH 225 ST service contract	$22,000 per year	Only if operating a CT scanner.
		Micro‐CT scanner, Nikon (Japan)
Tungsten filament replacements	Option 1N.1403: $299.99 for package of 10	Option 1: Ted Pella, Inc (California, USA)
*Recommended for Nikon XTH 225 ST	Option 2* A054X: $338 for package of 10	Option 2*: Agar Scientific (Essex, UK)
Iodine (crystalline)	$115.60 per 250 g	99.5%, Lot: Q26E019 Alfa Aesar. Thermo Fisher Scientific (MA, USA)
Potassium iodine	$299 per 100 g	Thermo Fisher Scientific (MA, USA)
Ethanol (EtOH)	$378.92 per 208.2 L drum	Thermo Fisher Scientific (MA, USA)
Packing peanuts	$29 per 20 ft 3 bag	Anti‐static, 30% recycled Uline (WI, USA)
Plastic jars	$00.28–$1.89 per jar	Clear round wide‐mouth plastic jars, Uline (WI, USA)
Soft packing foam	$61–$119 based on weight	Soft foam, Uline (WI, USA)
Poly tubing plastic	$16–$50 based on length and diameter ordered	Poly tubing plastic dispenser, Uline (WI, USA)
Technician/personnel	$8000–$65,000 per year	Cost of technician to operate a CT facility depends on employee status (i.e., full time, student, postdoc, and part time)
Scanning cost	$48 per hour	Cost at University of Michigan CT facilities. This cost may vary, and may include packing and set up of the specimen
Estimated cost of a single diceCT acquisition	$216 per specimen	Does not include staining, destaining, or any analysis. The average diceCT scan takes 3.5 hr, plus an additional 30 min before and after for set up and data processing

For data storage, consider the total number and type of scans that will be generated, as each diceCT datasets can be in excess of 20 GB. While external hard drives are easily accessible and allow for data mobility between workstations, they are prone to failure and easily damaged or lost. Data can also be stored on “cloud” based servers, but users must consider subscription costs and international privacy laws of these services. An alternative to cloud‐based storage is Redundant Array of Inexpensive Disks (RAID) that can allow multiple workstations to be networked to a central data hub. These options may be more secure and offer redundancy that external hard drives do not, but at increased cost and lower portability.

### 4.4 Challenges and opportunities of digital segmentation

One of the primary challenges of analyzing diceCT data is interpreting the overwhelming complexity of soft‐tissue anatomy. Upon opening µCT slice data in a segmenting software, users are inundated with the entirety of internal and external morphology. Successful segmentation is the key step that transforms raw CT scans into usable morphological and life history information, critical to the wide array of downstream research questions and education goals within ecology and evolutionary biology. Identifying and segmenting pertinent anatomical structures are complicated by the overlapping range of GV among internal anatomy, in addition to the already existing anatomical variation (e.g., shape, size, and cell types) and interaction (e.g., networks of blood vessels and nerves). We found that different segmentation tools and approaches were needed depending on the user's ROI. For example, the brain is a large, lobed structure with varying GV ranges depending on the lobe region, thus relying on a thresholding tool for defining a set GV range is ineffective. Given that the brain is encased in a cranium (in reptiles and birds), it is relatively discrete from other cephalic organs. This feature of neural anatomy allows the user to add “scaffolds” to the CT stack, creating a closely clipped box around the brain and preventing overflow of thresholds values with GV of adjacent tissues. This technique can be used for other discrete structures such as the retina inside the eye. Other anatomical structures can be made discrete under diceCT due to variation in density and therefore GV ranges, for example, intraocular lens, vomeronasal organs, heat pit membranes, and diet items.

A range of approaches and tools can be used for segmenting nondiscrete and/or finer‐scale and intricately shaped structures or networks of structures, such as nerves or blood vessels (Figure 8; Figure S2). Image enhancements can be performed in various software such as Fiji (Schindelin et al., 2012) and AVIZO (version 2020.1, Thermo Fisher Scientific, MA, USA) to make segmentations easier to complete. Alterations to enhance the boundaries between structures, such as a Gamma correction or “unsharp mask,” can make adjacent organs discrete and thus easier to segment using thresholding tools (see Zuiderveld, 1994). Similarly, identifying how the ROI interfaces with surrounding anatomy (both in the diceCT and skeletal scans) by switching back‐and‐forth between image enhanced and skeletal scans can help determine the boundaries between structures and orient users while segmenting ROIs. For example, segmentation of the venom delivery system (Figure 6) was aided by referencing traditional dissection of the original specimen and combining the skeletal and diceCT scans to find the connections between fang/maxilla, venom duct, and gland. This process was especially important for nonfront‐fanged species such as colubrines and dipsadines (Figure 7). Similarly, heat‐sensitive membranes and their associated nerves branching from the trigeminal ganglion were revealed in relation to foramina of the maxilla bone from the skeletal scan (Figure S2).

A great advantage of diceCT is the creation of digital specimens that allow multiple users to independently characterize and measure the same phenotype across many specimens. However, reproducibility of segmentation in diceCT scans should be tested to ensure repeatability of downstream morphological analyses (e.g., volume and shape measurements). Anecdotally, we found that segmentation variation among users was greatest when (a) poor staining/resolution quality of specimens, and (b) new users were unfamiliar with segmentation software and/or specimen anatomy. Ensuring that specimens are adequately stained and packed before CT scanning will ultimately result in easier segmentation for users. To help identify anatomical relationships and increase user familiarity with diceCT, we recommend “exploratory” sessions, whereby the user is exposed to multiple training sets of scans and is free to scroll through adjacent 2D tomography slices. Identifying large, adjacent morphological features, or structures can make great “reference points” during segmentation of diceCT scans. Access to taxonomic and anatomical descriptions of specimens are also invaluable reference materials (e.g., Gans, 1969‐2010; Taub, 1966; Underwood, 1967) and should be used in conjunction with 3D models. Despite this extensive literature, however, users experienced difficulty interpreting soft‐tissue data because of the complex interconnecting anatomy, overlapping GV ranges, and 3D planes of rotation. Discrepancy in segmentations was highest for the oral and cephalic glands of nonfront fanged colubrid snakes (Figures 6, 7). Glands from these snakes can vary in size, shape, location, textural appearance, and density (Jackson et al., 2017), as well as being influenced by staining quality. Generally, a combination of approaches (including traditional dissection) may be needed to identify boundaries, interfaces, and connections among internal anatomy (Figure 6).

### 4.5 Data curation and storage

Data curation is necessary for scientific reproducibility and compliance with institutional regulations (e.g., academic journals and funding bodies). Once scans are hosted online, anyone with an Internet connection can access morphological data that was historically inaccessible. There are a number of web‐based repositories to store data for this purpose such as Dryad, Morphosource, and DigiMorph. Data may also be archived in research institution libraries (see: UM Libraries Deep Blue Data). Derived µCT data objects (e.g., segmentations) may fall under the purview of creative commons licenses whereby the original author is credited for their work, but this is not yet an established practice. Finally, data sharing policies for diceCT should be internationally standardized to ensure data are accessible across educational and/or research institutions.

We recommend scanning the entire body and ROI of specimens for both traditional µCT and diceCT, especially for museum collections. This will ensure that specimens are only ever diceCT scanned once, thereby minimizing the potential effects of staining and destaining process, and providing future access to the entire ”digital specimen.” Data management plans should implement a standardized system for naming files to facilitate searching large datasets and data archives. Naming conventions should include details of museum and specimens tags, taxonomic identifier, and type of scan (stained or unstained; ROI), and be stored in a hierarchy of directories according to taxonomic rank. Data management plans must ensure that there is sufficient storage capacity for both processing and archiving data. Due to the size of the datasets, 3D rendering, and the complexity of the potential analyses that can be derived from the data, any workstation used will need to contain a higher random access memory (RAM) size (64‐126GB), a graphics processing unit (GPU) with dedicated memory (2–8 GB), and an up to date central processing unit (CPU).

### 4.6 Filaments for CT scanners

The lifespan of the filament should be factored into project timelines. The lifespan is dependent on the scanning parameters used, duration of scans, the quality of replacement and alignment, and cleanliness of the CT scanner. At the UMMZ, we use A054X filaments (Agar Scientific, Essex, UK) which typically last about 200 hr of scanning, and AEI style tungsten filaments No.1403 (Ted Pella Inc, California, USA) which were recommended by the Nikon CT scanner manufacturer. However, when our Agar supply was depleted our administrator opted for the Ted Pella Inc. brand, which was at a lower price point (Table 2). As a result, we have noticed a lowered filament lifespan to approximately 125 hr. While there is a benefit to saving by ordering equivalent filaments from other vendors, it is best to order the manufacturer’s recommended parts as it will be more cost effective in the long run.

### 4.7 Recommendations for future diceCT studies

DiceCT uncovers internal anatomy of largely inaccessible museum collections with minimal modification to the original specimen, revolutionizing the capacity for high‐throughput phenotyping across the tree of life. DiceCT is a powerful tool to quantify morphological variation, both intra‐ and interspecifically, and can be applied to a comparative phylogenetic framework (Figure 8; Macrì et al., 2019). A workflow that ensures both diceCT and skeletal CT scanning ensures a comprehensive digital specimen with access to morphological data and natural history bycatch (Figures 7, 8, 9). To ensure that diceCT data can be used in perpetuity and for the broadest range of research and educational applications, the longevity of both the digital and physical specimens should be prioritized. Generating µCT data are likely to become quicker and easier, resulting in a boom of digital specimens and technological advances to visualize finer‐detailed ultrastructure that previously required destructive techniques such as histology. Improvements to postscanning analysis are also likely to aid users in quickly filtering and segmenting ROIs (see Furat et al., 2019). In this way, diceCT may experience parallel issues to the Big Data generated by DNA sequencing technologies and subsequent lag in expertise to curate and analyze the glut of digital data.

DiceCT presents an unprecedented opportunity for analyses of phenotypic evolution and ecological diversification, as well as innovative educational and outreach resources for communicating science to a broader audience. As diceCT technology advances, we should invest in anatomical research that can provide resources of intra‐ and interspecific variation in anatomy (e.g., 3D visual atlas), as well as comprehensive training of morphologists and investing in open‐source software and data repositories.

## CONFLICT OF INTEREST

The authors state no conflicts of interest.

## AUTHOR CONTRIBUTION

**Sean Callahan:** Conceptualization (lead); Data curation (equal); Formal analysis (supporting); Investigation (lead); Methodology (lead); Project administration (equal); Visualization (equal); Writing‐original draft (equal); Writing‐review & editing (equal). **Jenna Margaret Crowe‐Riddell:** Conceptualization (lead); Data curation (equal); Formal analysis (lead); Investigation (supporting); Methodology (supporting); Project administration (supporting); Resources (supporting); Software (equal); Visualization (equal); Writing‐original draft (lead); Writing‐review & editing (lead). **Ramon S Nagesan:** Conceptualization (equal); Data curation (equal); Investigation (equal); Methodology (equal); Project administration (equal); Software (equal); Supervision (equal); Writing‐original draft (equal); Writing‐review & editing (equal). **Jaimi Gray:** Conceptualization (supporting); Investigation (equal); Methodology (equal); Resources (equal); Software (equal); Visualization (equal); Writing‐original draft (equal); Writing‐review & editing (equal). **Alison Davis Rabosky:** Conceptualization (lead); Data curation (equal); Formal analysis (supporting); Funding acquisition (equal); Investigation (supporting); Methodology (equal); Project administration (equal); Resources (equal); Supervision (equal); Validation (supporting); Visualization (equal); Writing‐original draft (equal); Writing‐review & editing (equal).

## Supporting information

Appendix S1‐3

## Data Availability

The DOIs for diceCT scans of snake heads are available in Table S2, and are available from Morphosource (https://www.morphosource.org/Detail/ProjectDetail/Show/project_id/374 ) and Sketchfab (https://sketchfab.com/michiganherpetology). R script to plot grayscale values and linear regression analyses available on github (https://github.com/jcroweriddell/guide‐diceCT‐snakes ).
