# Supplementary material for: A guide for optimal iodine staining and high‐throughput diceCT scanning in snakes
Source: Ecol Evol. 2021 Jun 26;11(17):11587–603. doi: 10.1002/ece3.7467 (PMC8427571; doi:10.1002/ece3.7467)
Supplement: Supplementary file 1 — Appendix S1‐3 [file ECE3-11-11587-s001.doc]

**Appendices**

**Appendix S1**: Recipe for 1.25% Lugol’s iodine ( I_2_ + KI) solution (1 L)

*Materials*

- 12.5 g Reagent grade iodine crystals
- 25 g Reagent grade potassium iodine crystals
- 1 L Deionized water (DI H_2_O)

*Steps*

1. Fill a container with 1.0 L of DI water.
2. Measure 12.5 g of iodine crystals.
3. Measure 25 g of potassium iodine crystals.
4. Mix the crystals into the DI H_2_O.
5. Shake the container well.
6. Add a mixing tab into the container.
7. Place mixture filled container onto mixing platform - set to maximum.
8. Allow to mix for up to 48 h.
9. Mix and store in a dark location. Prepare as needed.

**Appendix S2:** Scanning parameters and Morphosource media link to access scans.

**Table S1**. Scanning parameters used for specimens in this study. Scanner voltage (kV), scanner amperage (uA), exposure time (ms).

| **Clade** | **Taxon** | **Scan type** | **Voxel size mm** | **kV** | **uA** | **ms** | **Projections** | **Frame averaging** | |
| --- | --- | --- | --- | --- | --- | --- | --- | --- | --- |
| Aniliidae | *Anilius scytale* | Skeletal | 0.0377 | 85 | 200 | 250 | 1601 | | 2 |
|  |  | Skeletal_skull | 0.01655 | 85 | 120 | 250 | 1601 | | 2 |
|  |  | diceCT_skull | 0.01932 | 85 | 200 | 250 | 3141 | | 16 |
|  |  | diceCT | 0.05201 | 85 | 200 | 250 | 3141 | | 16 |
| Colubrinae | *Chironius fuscus* | Skeletal | 0.06869 | 85 | 200 | 250 | 1601 | | 2 |
|  |  | Skeletal_skull | 0.01777 | 85 | 200 | 250 | 1601 | | 2 |
|  |  | diceCT_skull | 0.02174 | 85 | 200 | 250 | 3141 | | 16 |
|  |  | diceCT | 0.0564 | 85 | 200 | 250 | 3141 | | 16 |
|  | *Pseustes sulphureus* | Skeletal | N/A | N/A | N/A | N/A | N/A | | N/A |
|  |  | Skeletal_skull | 0.04569 | 100 | 200 | 250 | 1601 | | 2 |
|  |  | diceCT_skull | N/A | N/A | N/A | N/A | N/A | | N/A |
|  |  | diceCT | 0.0468 | 140 | 80 | 250 | 3141 | | 8 |
|  | *Lampropeltis abnormal* | Skeletal | 0.04792 | 85 | 200 | 250 | 1601 | | 2 |
|  |  | Skeletal_skull | 0.02041 | 85 | 200 | 250 | 1601 | | 2 |
|  |  | diceCT_skull | 0.01843 | 85 | 200 | 250 | 3141 | | 16 |
|  |  | diceCT | 0.0797 | 85 | 200 | 250 | 3141 | | 16 |
|  | *Leptophis ahaetulla* | Skeletal | N/A | N/A | N/A | N/A | N/A | | N/A |
|  |  | Skeletal_skull | 0.01502 | 100 | 150 | 250 | 1601 | | 2 |
|  |  | diceCT_skull | 0.0157 | 85 | 185 | 250 | 3141 | | 8 |
|  |  | diceCT | N/A | N/A | N/A | N/A | N/A | | N/A |
|  | *Nothopsis capensis* | Skeletal | 0.03093 | 85 | 200 | 250 | 1601 | | 2 |
|  |  | Skeletal_skull | 0.0113 | 85 | 120 | 250 | 1601 | | 2 |
|  |  | diceCT_skull | 0.01289 | 85 | 120 | 250 | 3141 | | 16 |
|  |  | diceCT | 0.04912 | 85 | 200 | 250 | 3141 | | 16 |
|  | *Tantilla melanocephala* | Skeletal | N/A | N/A | N/A | N/A | N/A | | N/A |
|  |  | Skeletal_skull | 0.01001 | 100 | 100 | 250 | 1601 | | 2 |
|  |  | diceCT_skull | 0.01401 | 85 | 165 | 250 | 3141 | | 8 |
|  |  | diceCT | N/A | N/A | N/A | N/A | N/A | | N/A |
| Dipsidinae | *Imantodes cenochoa* | Skeletal | 0.04551 | 85 | 200 | 250 | 1601 | | 2 |
|  |  | Skeletal_skull | 0.02042 | 85 | 200 | 250 | 1601 | | 2 |
|  |  | diceCT_skull | 0.0175 | 85 | 200 | 250 | 3141 | | 16 |
|  |  | diceCT | 0.05102 | 85 | 200 | 250 | 3141 | | 16 |
|  | *Helicops angulatus* | Skeletal | 0.0388 | 85 | 200 | 250 | 1601 | | 2 |
|  |  | Skeletal_skull | 0.02172 | 85 | 200 | 250 | 1601 | | 2 |
|  |  | diceCT_skull | 0.02268 | 85 | 200 | 250 | 3141 | | 16 |
|  |  | diceCT | 0.0591 | 85 | 200 | 250 | 3141 | | 16 |
|  | *Helicops leopardinus* | Skeletal | 0.07715 | 85 | 200 | 250 | 1601 | | 2 |
|  |  | Skeletal_skull | 0.02025 | 85 | 200 | 250 | 1601 | | 2 |
|  |  | diceCT_skull | 0.02597 | 85 | 200 | 250 | 3141 | | 16 |
|  |  | diceCT | 0.08475 | 85 | 200 | 250 | 3141 | | 16 |
|  | *Leptodeira septentrionalis* | Skeletal | 0.04712 | 85 | 200 | 250 | 1601 | | 2 |
|  |  | Skeletal_skull | 0.02003 | 85 | 200 | 250 | 1601 | | 2 |
|  |  | diceCT_skull | 0.01979 | 85 | 200 | 250 | 3141 | | 16 |
|  |  | diceCT | 0.06512 | 85 | 200 | 250 | 3141 | | 16 |
|  | *Oxyrhopus melanogenys* | Skeletal | 0.02829 | 85 | 200 | 250 | 1601 | | 2 |
|  |  | Skeletal_skull | 0.01167 | 85 | 120 | 250 | 1601 | | 2 |
|  |  | diceCT_skull | 0.01203 | 85 | 120 | 250 | 3141 | | 16 |
|  |  | diceCT | 0.0544 | 85 | 200 | 250 | 3141 | | 16 |
|  | *Xenopholis scalaris* | Skeletal | N/A | N/A | N/A | N/A | N/A | | N/A |
|  |  | Skeletal_skull | 0.0111 | 100 | 100 | 250 | 1601 | | 2 |
|  |  | diceCT_skull | 0.01251 | 100 | 125 | 354 | 3141 | | 2 |
|  |  | diceCT | N/A | N/A | N/A | N/A | N/A | | N/A |
| Elapidae | *Micrurus lemniscatus* | Skeletal | N/A | N/A | N/A | N/A | N/A | | N/A |
|  |  | Skeletal_skull | 0.0135 | 90 | 150 | 250 | 1601 | | 2 |
|  |  | diceCT_skull | 0.01539 | 85 | 180 | 250 | 3141 | | 16 |
|  |  | diceCT | N/A | N/A | N/A | N/A | N/A | | N/A |
|  | *Micrurus nigrocinctus* | Skeletal | 0.04812 | 85 | 200 | 250 | 1601 | | 2 |
|  |  | Skeletal_skull | 0.02288 | 85 | 200 | 250 | 1601 | | 2 |
|  |  | diceCT_skull | 0.02073 | 85 | 200 | 250 | 3141 | | 16 |
|  |  | diceCT | 0.05464 | 85 | 200 | 250 | 3141 | | 16 |
|  | *Micrurus obscurus* | Skeletal | N/A |  |  |  |  | |  |
|  |  | Skeletal_skull | 0.01288 | 85 | 120 | 250 | 1601 | | 2 |
|  |  | diceCT_skull | 0.01441 | 95 | 150 | 250 | 3141 | | 16 |
|  |  | diceCT | N/A | N/A | N/A | N/A | N/A | | N/A |
|  | *Micrurus surinamensis* | Skeletal | 0.04312 | 85 | 200 | 250 | 1601 | | 2 |
|  |  | Skeletal_skull | 0.01609 | 85 | 120 | 250 | 1601 | | 2 |
|  |  | diceCT_skull | 0.01703 | 85 | 200 | 250 | 3141 | | 16 |
|  |  | diceCT | 0.05226 | 85 | 200 | 250 | 3141 | | 16 |
| Lamprophiidae | *Aparallactus capensis* | Skeletal | 0.01778 | 85 | 120 | 250 | 1601 | | 2 |
|  |  | Skeletal_skull | 0.010601 | 85 | 120 | 250 | 1601 | | 2 |
|  |  | diceCT_skull | 0.01071 | 85 | 120 | 250 | 3141 | | 16 |
|  |  | diceCT | 0.05116 | 85 | 200 | 250 | 3141 | | 16 |
|  | *Atractaspis bibronii* | Skeletal | 0.03406 | 85 | 200 | 250 | 1601 | | 2 |
|  |  | Skeletal_skull | 0.01192 | 85 | 120 | 250 | 1601 | | 2 |
|  |  | diceCT_skull | 0.0143 | 85 | 120 | 250 | 3141 | | 16 |
|  |  | diceCT | 0.04435 | 85 | 200 | 250 | 3141 | | 16 |
| Viperidae | *Bothrops bilineatus* | Skeletal | 0.05563 | 85 | 200 | 250 | 1601 | | 2 |
|  |  | Skeletal_skull | 0.0268 | 85 | 200 | 250 | 1601 | | 2 |
|  |  | diceCT_skull | 0.02537 | 85 | 200 | 250 | 3141 | | 16 |
|  |  | diceCT | 0.07185 | 85 | 200 | 250 | 3141 | | 16 |
|  | *Causus rhombeatus* | Skeletal | 0.04752 | 85 | 200 | 250 | 1601 | | 2 |
|  |  | Skeletal_skull | 0.01974 | 85 | 200 | 250 | 1601 | | 2 |
|  |  | diceCT_skull | 0.02976 | 85 | 200 | 250 | 3141 | | 16 |
|  |  | diceCT | 0.07015 | 85 | 200 | 250 | 3141 | | 16 |
|  | *Lachesis muta* | Skeletal | 0.04945 | 85 | 200 | 250 | 1601 | | 2 |
|  |  | Skeletal_skull | 0.03019 | 85 | 200 | 250 | 1601 | | 2 |
|  |  | diceCT_skull | 0.0338 | 85 | 200 | 250 | 3141 | | 16 |
|  |  | diceCT | 0.06944 | 85 | 200 | 250 | 3141 | | 16 |
|  | *Porthidium nasutum* | Skeletal | 0.04575 | 85 | 200 | 250 | 1601 | | 2 |
|  |  | Skeletal_skull | 0.01478 | 85 | 120 | 250 | 1601 | | 2 |
|  |  | diceCT_skull | 0.01317 | 85 | 120 | 250 | 3141 | | 16 |
|  |  | diceCT | 0.04594 | 85 | 200 | 250 | 3141 | | 16 |

**Table S2**. Morphosource media ID and links for specimens used in this study. UMMZ = University of Michigan Museum of Zoology, USA; MUSM = Museo de Historia Natural de la Universidad Nacional Mayor de San Marcos, Lima, Peru.

| **Genus** | **Mus** | **Number** | **Elements** | **Media ID** | **DOI** |
| --- | --- | --- | --- | --- | --- |
| *Anilius scytale* | UMMZ | 248356 | skull dice | M82104 | <https://doi.org/10.17602>/M2/M158796 |
| *Chironius fuscus* | UMMZ | 245047 | skull dice | M82090 | <https://doi.org/10.17602/M2/M168782> |
| *Pseustes sulphureus* | MUSM | 37565 | skull dice | M82214 | <https://doi.org/10.17602/M2/M159090> |
| *Lampropeltis abnormal* | UMMZ | 247095 | skull dice | M82100 | <https://doi.org/10.17602/M2>/M158792 |
| *Leptophis ahaetulla* | MUSM | 37345 | skull dice | M82211 | <https://doi.org/10.17602/M2/M159087> |
| *Tantilla melanocephala* | UMMZ | 246845 | skull dice | M82118 | <https://doi.org/10.17602/M2>/M158824 |
| *Imantodes cenchoa* | UMMZ | 246810 | skulldice | M86059 | <https://doi.org/10.17602/M2/M167281> |
| *Helicops angulatus* | UMMZ | 246805 | skull | M82093 | <https://doi.org/10.17602/M2>/M158785 |
|  |  |  | skull dice | M82094 | <https://doi.org/10.17602/M2>/M158786 |
| *Helicops lepardinus* | UMMZ | 246808 | skull dice | M82095 | <https://doi.org/10.17602/M2/M158787> |
| *Leptodeira septentrionalis* | UMMZ | 247099 | skeletal | M82101 | <https://doi.org/10.17602/M2/M158793> |
|  |  |  | dice body | M82102 | <https://doi.org/10.17602/M2>/M158794 |
| *Nothopsis rugosus* | UMMZ | 248404 | skull dice | M82106 | <https://doi.org/10.17602/M2>/M158798 |
| *Oxyrhopus melanogenys* | MUSM | 37417 | skull dice | M82213 | <https://doi.org/10.17602/M2/M159089> |
| *Xenopholis scalaris* | UMMZ | 246854 | skull dice | M82097 | <https://doi.org/10.17602/M2/M158789> |
| *Micrurus lemniscatus* | MUSM | 35905 | skull dice | M82210 | <https://doi.org/10.17602/M2/M159086> |
| *Micrurus nigrocinctus* | UMMZ | 247142 | skull dice | M82103 | <https://doi.org/10.17602/M2>/M159795 |
| *Micrurus obscurus* | UMMZ | 246859 | skull dice | M82099 | <https://doi.org/10.17602/M2/M158791> |
| *Micrurus surinamensis* | MUSM | 37353 | skull dice | M82212 | <https://doi.org/10.17602/M2>/M159088 |
| *Aparallactus capensis* | UMMZ | 61599 | skul dice | M82107 | <https://doi.org/10.17602/M2/M158799> |
| *Atractaspis bibronii* | UMMZ | 209986 | skull dice | M82109 | <https://doi.org/10.17602/M2/M158804> |
| *Bothrops bilineatus* | UMMZ | 245084 | skull dice | M82092 | <https://doi.org/10.17602/M2/M158784> |
| *Lachesis muta* | UMMZ | 248369 | skull dice | M82105 | <https://doi.org/10.17602/M2/M158797> |
| *Causus rhombeatus* | UMMZ | 65828 | skulldice | M86620 | <https://doi.org/10.17602/M2/M167972> |
| *Porthidium nasutum* | UMMZ | 247139 | skulldice | M86619 | <https://doi.org/10.17602>/M2/M167971 |

**Appendix S3 Supplementary images**


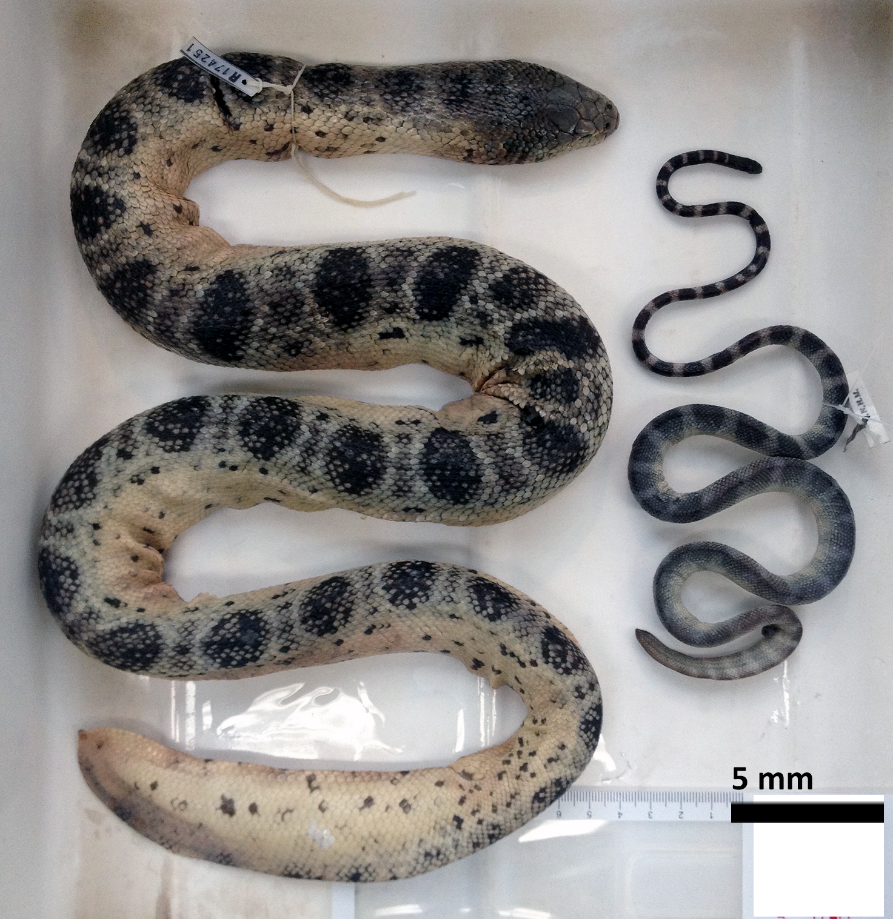


**Figure S1**. Example of alternative preservation position for snakes to make packing and CT scanning easier. Snakes are typically packed in a tight coil to fit into preservation jars.


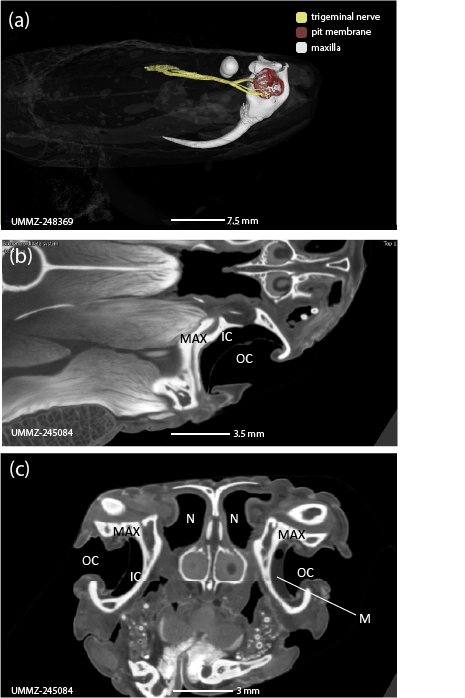


**Figure S2.** DiceCT of heat-pit system in vipers. (a) 3D render of a heat-pit membrane and associated trigeminal nerve branches relative to the maxilla bone in *Lachesis muta* (UMMZ 248369). Eyes are rendered in white for positional reference. 2D tomography slices show anatomy of the heatpit in *Bothriopsis bilineata* (UMMZ 245084) in (b) frontal, and (c) transverse. MAX = maxilla, M = membrane, N = nare, OC = outer cavity, IC = inner cavity.
